# Controlled Self‐Assembly and Photo‐Thermal Activation of Viologen‐Based 2D Semiconductors for Dual‐Function Energy Management in All‐Weather Applications

**DOI:** 10.1002/advs.202415101

**Published:** 2025-02-08

**Authors:** Muhammad Sultan Irshad, Iftikhar Ahmed, Naila Arshad, Muneerah Alomar, Yue Shu, Guo Zhenzhen, Shafiq Ahmad, Ioannis Zuburtikudis, Shi Ruiqing, Tao Mei, Fan Xiaochao, Nang Xuan Ho, Thuy‐Duong Pham, Van‐Duong Dao, Rong Li, Xianbao Wang

**Affiliations:** ^1^ Ministry‐of‐Education Key Laboratory for Green Preparation and Application of Functional Materials School of New Energy and Electrical Engineering Hubei University Wuhan 430062 P. R. China; ^2^ Environmental and Public Health Department College of Health Sciences Abu Dhabi University P.O. Box 59911 Abu Dhabi UAE; ^3^ State Key Laboratory of Advanced Technology for Materials Synthesis and Processing, School of Materials Science and Engineering Wuhan University of Technology Wuhan 430070 P.R. China; ^4^ Department of Industrial Engineering College of Engineering King Saud University Riyadh 11564 Saudi Arabia; ^5^ Department of Chemical Engineering Abu Dhabi University P.O. Box 59911 Abu Dhabi UAE; ^6^ Xinjiang Institute of Engineering Urumqi Xinjiang 830047 P.R. China; ^7^ Faculty of Vehicle and Energy Engineering Phenikaa University Hanoi 100000 Vietnam; ^8^ Faculty of Biotechnology, Chemistry, and Environmental Engineering Phenikaa University Hanoi 100000 Vietnam; ^9^ School of Intelligent Manufacturing Hubei University Wuhan 430062 China

**Keywords:** 2D, electrothermal, energy generation, evaporation, Mn^II^–Fe^III^@CM, photothermal, viologen

## Abstract

Solar thermal technology offers a promising solution to water scarcity; however, the continuous operation of solar evaporators remains challenging due to sunlight's intermittent availability. Herein, an alternative strategy is proposed to achieve dual‐functional energy management of photo‐thermoactivated viologen T semiconductors for enhanced solar water evaporation, water‐enabled electricity generation, and electrothermal evaporation. A sequential cyanide‐bridged layer‐directed intercalation approach is developed, where infinitely π‐stacked, redox‐active N‐methyl bipyridinium cations with near‐planar structures are sandwiched between cyanide‐bridged Mn^II^–Fe^III^. The extended absorption range of 95% is achieved through radical–π interactions that occur within the continuously π‐stacked N‐methyl bipyridinium units upon thermal activation. The photo‐thermoactivated Mn^II^–Fe^III^ compounds anchored charcoal mask (Mn^II^–Fe^III^@CM) with a sided evaporation structure and controllable water transfer, offering a high evaporation rate of 2.39 kg m^−2^ h^−1^ under one sun (1 kW m^−2^) illumination. As an energy nanogenerator, the output voltage and current of Mn^II^–Fe^III^@CM can reach up to ≈480 mV and ≈60 µA cm^−2^ under ambient conditions. Furthermore, storage of electrical energy from Mn^II^–Fe^III^@CM using energy storage devices is expected to enable all‐weather evaporation by electric heating due to unsustainable sunlight, providing a unique technology for seawater desalination and offshore work platform energy access.

## Graphical abstract

An alternative strategy is proposed to achieve dual‐functional energy management of photo‐thermo‐activated viologen 2D semiconductors for enhanced solar water evaporation (2.39 kg m^−2^ h^−1^ under one sun), water‐enabled electricity generation (≈480 mV and ≈60 µA cm^−2^), and electrothermal evaporation approach is integrated to enable all‐weather evaporation due to unsustainable sunlight.

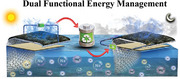

## 1 Introduction

Water shortage is a significant and urgent global issue that impacts millions of people and ecosystems on a global scale. The increasing need for freshwater resulting from population increase, industrialization, and agricultural requirements has placed a significant strain on water supplies.^[^ ^1^, ^2^, ^3^ ^]^ It is expected that 33–50% of the global urban population will face freshwater resource shortages by 2050. The abundant seawater reserves make seawater desalination technology an important way to solve the shortage of freshwater resources. At present, seawater desalination is mainly achieved through methods such as multi‐stage flash distillation, multi‐effect distillation, reverse osmosis, electrodialysis, membrane distillation, forward osmosis, etc.^[^ ^4^, ^5^, ^6^ ^]^ Among them, reverse osmosis, multi‐stage flash distillation, and multi‐effect distillation are currently the main commercial technologies. However, these technologies require pressurization of fossil fuels, natural gas, or electricity, commonly referred to as “oil for water” or “electricity for water”.^[^ ^7^, ^8^, ^9^, ^10^, ^11^ ^]^ Meanwhile, traditional seawater desalination technology often accompanies the emission of a large amount of greenhouse gases, resulting in serious environmental pollution. Facing the shortage of fossil fuels and environmental pollution around the world, utilizing renewable energy for seawater desalination has become a future development trend.^[^ ^12^, ^13^, ^14^ ^]^ Solar‐driven interfacial evaporation technology utilizes a solar evaporator to absorb sunlight and convert it into heat, locally heating the surface of seawater, rapidly evaporating cold water, and producing clean water.^[^ ^15^, ^16^, ^17^, ^18^ ^]^ This technology has enormous research value and broad application prospects with the characteristics of simple equipment and no pollution.

At present, the efforts made by researchers to improve the performance of solar water evaporation are mainly reflected in two aspects: the micro control of photothermal materials and the macro design of photothermal systems. The properties of photothermal materials determine their light absorption and photothermal conversion performance.^[^ ^19^, ^20^, ^21^, ^22^ ^]^ Photothermal materials with excellent light capture and photothermal conversion performance are an ideal choice for improving solar thermal evaporation performance from the source.^[^ ^23^, ^24^, ^25^, ^26^ ^]^ At present, scholars have conducted extensive research on enhancing photothermal conversion performance through micro/nanostructure design or bandgap control.^[^ ^27^, ^28^, ^29^, ^30^, ^31^, ^32^ ^]^ Viologen (*N*, *N*′‐disubstituted bipyridinium) based 2D semiconductor compounds have been shown as good candidates for broadband photoresponse.^[^ ^33^ ^]^ The strong cation‐π interactions between viologen components favor the construction of organic semiconductors, conductance and photoconductance of viologen‐based semiconductors may dramatically increase the photoinduced electron transfer (PET) and generation of free radical products. Secondly, a single viologen cation usually has a red‐shifted absorption band after forming a radical species. When viologen radicals are further closely π‐stacked, radical–π interactions that are stronger than cation–π, and π–π interactions will make the energy gap narrower and correspondingly absorption band much broader.^[^ ^34^ ^]^

The macro design focus of the photothermal system is to achieve a high evaporation rate and efficient desalination. The multi‐functional photothermal system designed by researchers is mainly to load photothermal materials on the membrane substrate, or porous sponge, or mix with organic matter to obtain a porous gel structure.^[^ ^13^, ^35^, ^36^, ^37^ ^]^ The acceleration of water transport by porous structure can promote multi‐directional diffusion, achieve salt ion convection, and to some extent solve the problem of salt deposition on the surface of the evaporator. Meanwhile, the inherent 3D structure of porous evaporators can increase the evaporation area and achieve high‐speed evaporation performance, which promotes the research of porous evaporation systems. However, there are still two problems to be solved: 1) Porous gel‐based evaporator is immersed in water for a long time, and the structure is easy to collapses; 2) Due to the unsustainable sunlight, the photothermal system lacks a heat source supply when there is no light, and the interface temperature of the photothermal device rapidly decreases, making the system unable to exert effective evaporation performance.

Charcoal Mask (CM), a material with good mechanical properties, is easy to fold and cut into any shape. By loading photothermal materials onto it and regulating the evaporation system (water transport and evaporation structure design), it is expected to achieve high‐rate evaporation and self‐desalination. There is no structural collapse during long‐term use, which provides a solution to the first problem. In addition, it is worth noting that the potential of solar photothermal technology has extended beyond independent functions. It can be integrated with other energy sources, including chemical gradient energy, mechanical energy, waste heat, and evaporation heat, opening the way for the establishment of innovative water resources‐cogeneration systems, and providing a promising optimization method for the utilization efficiency of water resources and energy. Among them, fluid flow‐induced power generation behavior, achieving synchronous water evaporation and power generation performance, has become one of the up‐and‐coming methods. Therefore, future efforts may focus on exploring new integrated systems that combine solar evaporation and power generation, which provides a strong strategy for solving the second problem.

**Figure 1:**
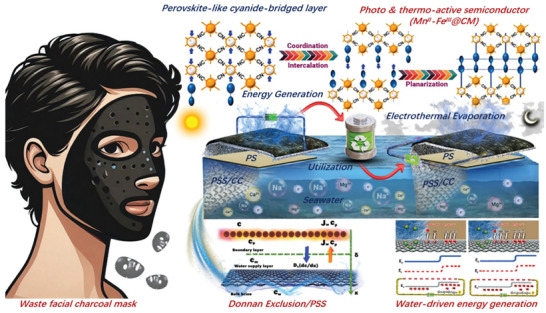
Schematic illustration of dual functional energy management achieved by viologen 2D semiconductor is developed by cyanide‐bridged layer‐directed intercalation approach for all‐weather utilization.

In this work, an alternative strategy is proposed to achieve dual‐functional energy management of photo‐thermoactivated viologen 2D semiconductors for enhanced solar water evaporation, water‐enabled electricity generation, and electrothermal evaporation, as illustrated in **Figure** **1**. A sequential cyanide‐bridged layer‐directed intercalation approach is developed, where infinitely π‐stacked, redox‐active N‐methyl bipyridinium cations with near‐planar structures are sandwiched between cyanide‐bridged Mn^II^–Fe^III^. The extended absorption range of 95% is achieved through radical–π interactions that occur within the continuously π‐stacked N‐methyl bipyridinium units upon thermal activation. The photo‐thermoactivated Mn^II^–Fe^III^ compounds anchored charcoal mask (Mn^II^–Fe^III^@CM) with a sided evaporation structure and controllable water transfer, offering a high evaporation rate of 2.39 kg m^−2^ h^−1^ under one sun (1 kW m^−2^) illumination. As an energy nanogenerator, the output voltage and current of Mn^II^–Fe^III^@CM can reach up to ≈480 mV and ≈60 µA cm^−2^ under ambient conditions. The maximum voltage can be further increased to ≈640 mV cm^−2^ under one sun illumination. Furthermore, storage of electrical energy from Mn^II^–Fe^III^@CM using energy storage devices is expected to enable all‐weather evaporation by electric heating due to unsustainable sunlight. The study investigates Mn^II^–Fe^III^@CM for freshwater and electricity generation, providing a unique technology for saltwater desalination and offshore work platform energy access.

## 2 Results and Discussion

### 2.1 Structural and Morphological Analysis

**Figure 2:**
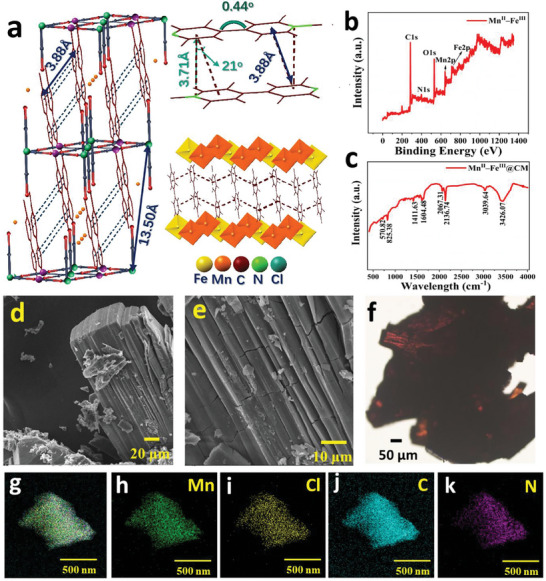
a) Crystal structure of 1. (**Left)** Side view of the 3‐D packing structure; (**Right)** two π‐stacked MQ^+^ cations; c infinitely π‐stacked MQ^+^ cations between two perovskite‐like cyanide‐bridged layers (cyano groups are drawn as vertexes of octahedra). b) XRD pattern of Mn^II^–Fe^III^. c) FTIR spectra of Mn^II^–Fe^III^. d,e) FESEM image of 2D Mn^II^–Fe^III^ showing stacked 2D layers. f) Inverted confocal microscopic image of 2D Mn^II^–Fe^III^. g–k) HRTEM images and EDS mapping of the of 2D Mn^II^–Fe^III^ showing the elemental conposition.

Single crystals of [{Mn^II^(MQ)_2_}{Fe^III^(CN)_6_}]Cl·3H_2_O was obtained from the diffuse reaction of MnCl_2_·5H_2_O, MQCl·H_2_O, and K_3_[Fe^III^(CN)_6_] in a molar ratio of 1:2:1 in water. The X‐ray diffraction (XRD) pattern of the Mn^II^–Fe^III^ is shown in Figure S1 (Supporting Information). The XRD analysis demonstrates a highly crystalline nature in its original crystalline phase. The sharp and well‐defined peaks in the XRD pattern indicate its ordered crystal structure, suggest the regular atomic arrangement, and match the previously reported data well.^[^ ^33^ ^]^ However, the process of grinding leads to a reduction in crystallinity and leads transition to a more disordered or amorphous phase, which is reflected in the XRD patterns as a decrease in the intensity and sharpness of the peaks which can enhance the material's surface area, and increase its interaction with light, which improves solar absorption and create more sites for light absorption and potentially enhance thermal energy conversion efficiency. As can be seen from **Figure** **2**a, cyanide‐bridged layers of Mn^II^–Fe^III^ are intercalated through π‐π and cation‐π interactions between two adjacent MQ^+^ ligands. Each Fe^III^ atom coordinates to six cyano groups (four, bridged, two, mono‐coordinated), while each Mn^II^ atom is ligated by two MQ^+^ ligands and four bridged cyano groups from four [Fe(CN)_6_]^3−^ units (Left). The interannular angle of each MQ^+^ cation is 0.44° (Right), close to being planar. Centroid (pyridyl)‐centroid (pyridyl) and N‐centroid (pyridyl) distances between two adjacent π‐stacked MQ^+^ cations are 3.88 and 3.71 Å, respectively. Every π‐stacked MQ^+^ layer is sandwiched by two cyanide‐bridged layers, which offers a chance to shield the air.^[^ ^33^ ^]^ The elemental composition and chemical states analysis of the Mn^II^–Fe^III^ was carried out by performing the XPS (X‐ray Photoelectron Spectroscopy) which reveals the presence of C1s, N1s, O1s, Mn2p, and Fe2p core levels, as shown in Figure 2b. The high‐resolution spectrum of C1s, Mn2p, and Fe2p are shown in Figures S2–S4 (Supporting Information). The C1s spectrum shows two distinct peaks, one at 284.82 eV corresponding to carbon atoms in C‐C or C‐H bonds, likely from the viologen (MQ) rings. The second peak at 286.68 eV represents carbon atoms in C‐N bonds, likely from the cyanide (CN) ligands or the nitrogen‐containing structure of viologen. In the Mn2p high‐resolution spectrum, two main peaks are observed at 641.6 eV (Mn 2p_3/2_) and 653.5 eV (Mn 2p_1/2_), consistent with the Mn^II^ oxidation state. The 641.6 eV peak is deconvoluted into four subpeaks at 641.1, 641.5, 642.2, and 642.9 eV, indicating different local environments or slight variations in the electronic structure of Mn^II^ within the lattice. The second peak at 653.5 eV is deconvoluted into three subpeaks at 653.04, 653.55, and 653.98 eV, further confirming the presence of Mn^II^ with complex electronic interactions in the material. The Fe2p spectrum shows a peak around 711 eV, which is characteristic of Fe^III^ in the Fe^III^(CN)_6_ unit, accompanied by satellite peaks around 719 eV, indicating the high‐spin Fe^III^ state. Further, the FTIR spectrum of Mn^II^–Fe^III^ displays several notable peaks indicative of various functional groups, as shown in Figure 2c. The peak at 570.82 cm^−1^ is likely associated with Mn–O or Fe–O stretching vibrations. Peaks at 825.38 and 1416.3 cm^−1^ are typically attributed to C─N stretching vibrations in the viologen rings and cyanide groups. The strong peaks at 1604.48 and 2067.31 cm^−1^ correspond to C═C stretching and C≡N stretching vibrations, respectively, reflecting the presence of viologen and cyanide groups. The peak at 2136.74 cm^−1^ further confirms the presence of C≡N stretches. The broad peaks at 3039.64 and 3426.07 cm^−1^ are characteristic of O–H stretching vibrations from the water of hydration, highlighting the role of water molecules in the structure. The FESEM images of the viologen‐based 2D semiconductor reveal a distinct layered structure, with flat, and stacked sheets exhibiting a well‐defined 2D morphology. The high‐resolution images show these layers are uniform and extend over large areas, indicative of the successful synthesis of a 2D material. The clear, smooth surface and consistent layering highlight the material's potential for applications where a well‐ordered 2D structure is crucial (Figure 2d,e). Further, Mn^II^–Fe^III^ was also examined by inverted confocal microscopy. Figure 2f shows the inverted confocal microscopic image illustrates a 2D arrangement of a Mn^II^–Fe^III^. The observed dark and reddish areas signify the layered morphology characteristic with planar arrangement, which promotes interlayer interactions. Reversible changes in the electronic structure of the Mn^II^‐Fe^III^ under light exposure, most likely involving metal‐to‐metal or ligand‐to‐metal charge transfer processes, are the source of the photochromic behavior. The HRTEM EDS mapping images illustrate the elemental distribution within a 2D Mn^II^–Fe^III^ semiconducting material, offering a comprehensive visualization of its composition and structure at the nanoscale. Figure 2g illustrates the overall morphology and elemental distribution, demonstrating a homogeneous structure. Figure 2g–k illustrate the consistent distribution of essential elements (Mn, Cl, C, and N) within the 2D Mn^II^–Fe^III^ semiconducting material, affirming its structural integrity and elemental uniformity. The consistent distribution of elements is crucial for preserving the material's semiconducting characteristics and its prospective uses in electronic and optoelectronic devices. Figure S6 (Supporting Information) represents EDS spectra of 2D Mn^II^–Fe^III^ semiconducting material, and the detailed composition is depicted in Table S1 (Supporting Information).

**Figure 3:**
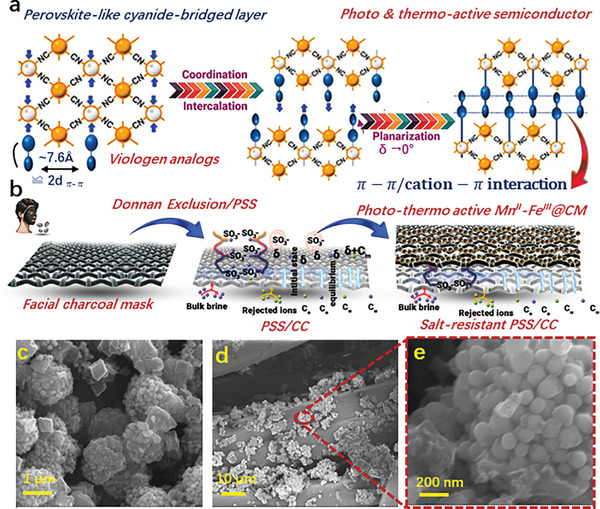
a) Design strategy in this work. δ and d_π–π_ denote the interannular angle and common separation for π–π interactions, respectively. b) Schematic illustration for the synthetic process of Mn^II^–Fe^III^@CM. FESEM images of c) Pyrolyzed Mn^II^–Fe^III^ and d,e) Pyrolyzed Mn^II^–Fe^III^ deposited over the charcoal mask.

A cyanide‐bridged layer‐directed intercalation method is adopted to achieve all the mentioned objectives and produce single‐component viologen‐based semiconductors with intrinsic UV–SWIR photoresponse capabilities, as well as photo/thermo activity and extended lifetimes for radical products. Each cyanide‐bridged layer in Prussian blue or its analogs with perovskite‐like structures features periodically arranged hexacoordinated metal sites and limited metal‐to‐metal distances (**Figure** **3**a). The periodic arrangement of these metal coordination sites promotes the orderly and continuous accumulation of axial ligands. The closest non‐contact metal‐to‐metal distances, typically around 7.6 Å, are ideal for supporting two π‐stacking interactions.^[^ ^33^ ^]^ As schematically illustrated, a sandwiched inorganic‐organic hybrid structure with infinitely stacked organic supramolecular layers will be formed, when metals with these distances are all coordinated by one viologen ligand or its analogs and then the layers are intercalated. The coexistence of π‐π and cation‐π interactions in the organic supramolecular layer is predictable since viologen and its analogs are aromatic cations.^[^ ^33^ ^]^ As mentioned above, this case favors the construction of a semiconductor. In addition, the viologen ligand or its analogs will become planarization owing to the close stacking of adjacent ligands, which may bring thermo activeness as stated above. Based on these considerations, we integrated the redox photoactive *N*‐methyl bipyridinium (MQ^+^) cation into cyanidebridged Mn^II^–Fe^III^ layers as axial ligands and obtained a series of two viologen‐based 2D semiconductors, [{Mn^II^(MQ)_2_}{Fe^III^(CN)_6_}]Cl·3H_2_O. These semiconductors are both thermal and light active in the crystalline state.^[^ ^33^ ^]^ After PET, they generate long‐lived radical products and show intrinsic photoresponsive bands covering the UV–SWIR region (at least 355–2400 nm).^[^ ^33^ ^]^ The photothermal interfacial layer was prepared by coating Mn^II^–Fe^III^ on the waste facial charcoal mask. This photothermal layer was placed on the polystyrene sodium sulfonate (PSS) treated carbon cloth which is enriched with SO_3_^–^ groups facilitating the efficient salt rejection performance, which was applied to solar desalination (Figure 3b). This approach bestows balanced water transport, and the confined counter–ion Na^+^ induced the Donnan potential, which redistributed neighboring ions in the brine solution, thus enabling long‐term salt rejection. The FESEM images of the pyrolyzed Mn^II^–Fe^III^ reveal a granular‐type morphology, where the particles are agglomerated, forming a complex and interconnected structure showing significant thermal decomposition and restructuring, leading to the formation of tightly packed clusters. The surface appears rough with varying particle sizes, indicating non‐uniform grain growth (Figure 3c). The FESEM image of these particles deposited on the facial charcoal mask shows a more uniform distribution, resulting in a rough texture (Figure 3d). This roughness enhances light trapping and surface area, which in turn improves solar absorption efficiency. The magnified image reveals the average size of particles ～100 nm which contributes to increased surface area, promoting more catalytic sites for solar energy harvesting (Figure 3e). This uniform deposition and surface roughness enhance the material's optical properties, making it suitable for use in solar energy applications. The stress‐strain curve illustrates the mechanical properties of the 2D Mn^II^–Fe^III^@CM as shown in Figure S5 (Supporting Information). The graphs suggest that Mn^II^–Fe^III^@CM demonstrates higher strength (235 KPa) and enhanced ductility (35% strain) which denotes the material's ultimate tensile strength. This behavior illustrates the composite's capacity to endure substantial mechanical deformation without failure, which is crucial for durability and stability during operational cycles.

### 2.2 Solar Water Evaporation

**Figure 4:**
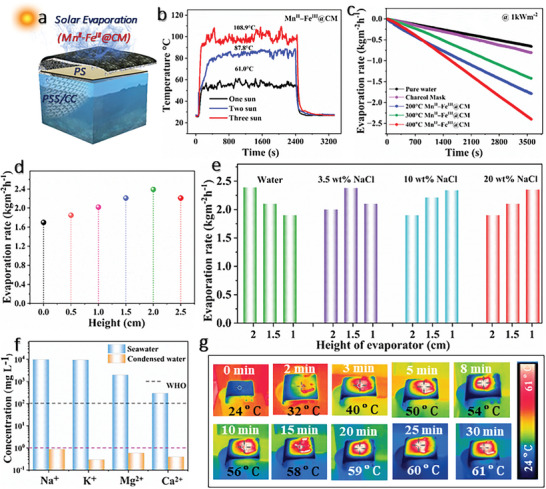
a) Schematic diagram of Mn^II^–Fe^III^@CM solar evaporator. b) Surface temperatures of Mn^II^–Fe^III^@CM under multiple solar intensities. c) Evaporation rates of developed five systems under one sun illumination. d) Evaporation rate of Mn^II^–Fe^III^@CM with different heights under one sun illumination. e) Evaporation rates under different brine concentrations of Mn^II^–Fe^III^@CM with different heights. f) The performance against primary metal ions. g) Time‐dependent infrared thermal imaging of Mn^II^–Fe^III^@CM under one sun illumination.

Photo‐thermoactivated semiconducting materials enhance solar water evaporation systems by efficiently converting sunlight into heat, improving energy efficiency and evaporation rates. To compare with traditional 2D evaporation systems, a double‐sided evaporation system with an air insulation layer (0.0267 W m^−1^ K^−1^) was designed to achieve a high evaporation area and reduce heat loss (**Figure** **4**a). High light absorption is a prerequisite for achieving efficient photothermal evaporation performance. Figure S7 (Supporting Information) shows the UV–vis absorption spectrum of the 400 °C treated Mn^II^–Fe^III^ over the whole solar spectrum from 200–2500 nm. The light absorption performance of Mn^II^–Fe^III^ can reach over 95% in the entire solar spectrum. The theoretical discussion of photothermal conversion efficiency and associated heat losses are explained in Note S1 (Supporting Information). The thermal conductivity of charcoal mask and Mn^II^–Fe^III^@CM was checked in wet state as shown in Figures S8 and S9 (Supporting Information), respectively (charcoal mask (0.05425 ± 0.00468 Wm^−1^ K^−1^) and Mn^II^–Fe^III^@CM (0.0997 ± 0.00636 Wm^−1^ K^−1^)) which are much lower than thermal conduction of water highlighting the good thermal management of system. Based on high light absorption and good thermal management, the surface temperature of Mn^II^–Fe^III^@CM under one sun was further measured under multiple solar intensities as shown in Figure 4b. The uniform deposition of the semiconductor particles on the mask, combined with the rough surface texture, plays a crucial role in trapping more sunlight, thereby accelerating heat generation. An increasing trend in surface temperature has been observed under higher solar intensity. Under 3 sun solar intensity, the temperature increases even more rapidly, reaching higher values compared to the lower intensities and the maximum temperature achieved is up to 108.9 °C under 3 kWm^−2^. The prepared Mn^II^–Fe^III^@CM solar evaporator was compared with other developed evaporation systems, i.e., pure water, charcoal mask, 200 °C Mn^II^–Fe^III^@CM, and 300 °C Mn^II^–Fe^III^@CM in terms of evaporation rate under one sun intensity for one hour. As expected, compared with other systems, 400 °CM Mn^II^–Fe^III^@CM exhibits better evaporation performance with a higher evaporation rate of 2.399 kg m^−2^ h^−1^ than that of water (0.65 kg m^−2^ h^−1^), charcoal mask (0.80 kg m^−2^ h^−1^), 200 °C Mn^II^–Fe^III^@CM (1.41 kg m^−2^ h^−1^) and 300 °C Mn^II^–Fe^III^@CM (1.75 kg m^−2^ h^−1^) (Figure 4c). The influence of the height of the air insulation layer on the photothermal evaporation performance has been studied. The heights of the air insulation layer are set to 0.5, 1, 1.5, 2, and 2.5 cm, respectively. During the adjustment process, the solar intensity at the evaporation interface is always maintained at 1 kW m^−2^. After one hour of light exposure, the evaporation rates of developed systems with 0, 0.5, 1, 1.5, 2, and 2.5 cm air insulation layers are ≈1.7, ≈1.85, ≈2.02, ≈2.21, ≈2.39, and ≈2.21 kg m^−2^, respectively (Figure 4d). It is evident that when the air insulation layer is 2 cm, the evaporation system achieves the highest evaporation performance, with an evaporation rate of up to ≈2.39 kg m^−2^ h^−1^. This is mainly because the double‐sided evaporation system increases the evaporation area and accelerates the evaporation performance, resulting in more heat being carried away due to evaporation. The saline water evaporation performance test of Mn^II^–Fe^III^@CM (Figure 4e) shows that for 3.5 wt% NaCl solution, the evaporation system achieves the highest evaporation rate (≈2.37 kg m^−2^ h^−1^) when the air insulation layer was 1.5 cm. When the concentration of NaCl solution increased to 10 and 20 wt.%, the evaporation system achieved the highest evaporation rate at an air insulation layer of 1 cm, which is ≈2.35 and ≈2.34 kg m^−2^ h^−1^, respectively. Experiments have shown that the height of the insulation layer that achieves the highest evaporation performance varies depending on different solutions, mainly constrained by water evaporation, thermal management, and water transport. Subsequently, Mn^II^–Fe^III^ solar evaporators were applied for the desalination of saline water. As shown in Figure 4f, the concentrations of Na^+^, Mg^2+^, Ca^2+^, and K^+^ decrease significantly with ion reject more than 99% and meeting the WHO standard of portable water, further suggesting promising application prospects in the field of seawater desalination. The time‐dependent Infrared (IR) images of Mn^II^–Fe^III^ solar evaporators under one sun intensity are shown in Figure 4g. Initially, the temperature starts at ambient levels and progressively increases as the material continues to absorb energy. Over time, the temperature rises steadily, reaching up to 61 °C, making the Mn^II^–Fe^III^‐based semiconductor an effective material for solar‐driven water treatment technologies.

### 2.3 Dual Functional Energy‐Steam Generator

**Figure 5:**
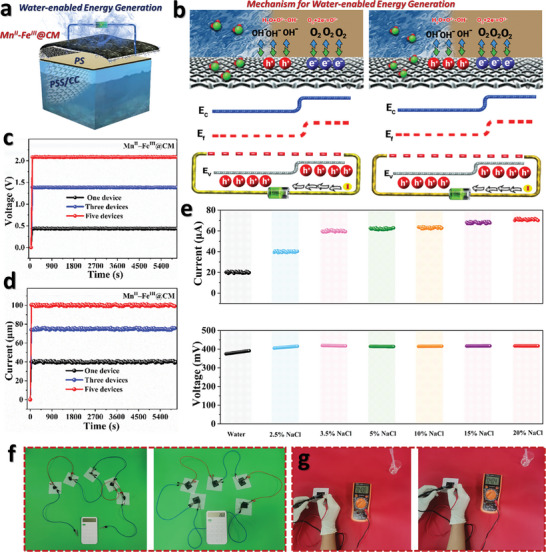
a) Schematic diagram of water‐driven energy generation. b) Mechanism analysis of water‐driven energy generation for Mn^II^–Fe^III^@CM. c) Voltage output performance and d) current output performance of the Mn^II^–Fe^III^@CM series connection. e) Voltage output performance and current output performance of Mn^II^–Fe^III^@CM by dropping different solutions. f,g) The output voltages under ambient conditions by connecting several devices in series with no solar irradiation.

A schematic diagram of the water‐driven energy generation process of Mn^II^–Fe^III^@CM is depicted in **Figure** **5**a. The device was connected to copper electrodes at both ends and outputs voltage and current during the transmission of water through capillary action, which were recorded by the acquisition system. Figure 5b shows a potential mechanism of hydroelectric power generation through Mn^II^–Fe^III^@CM. During the preparation process of Mn^II^–Fe^III^, hydrogen atoms can form covalent bonds. After contact with water, these hydrogen ions easily dissociate from the surface of Mn^II^–Fe^III^@CM into free‐hydrated hydrogen ions. Due to the uneven distribution of water, more hydrogen ions dissociate from the high humidity zone into free hydrated hydrogen ions, forming a concentration gradient of hydrated hydrogen ions with the low humidity zone, resulting in a potential difference. The energy generation of Mn^II^–Fe^III^@CM is determined by the gradient of hydrated hydrogen ions and changes in surface energy bands. On the one hand, after contact with water, hydrogen ions easily dissociate from the surface of Mn^II^–Fe^III^ into free hydrated hydrogen ions, resulting in potential differences due to asymmetric structure. On the other hand, in the air, oxygen molecules adsorbed on the surface of Mn^II^–Fe^III^ combine with electrons to form oxygen ions, increasing the concentration of charge carriers (holes) and causing the energy band to bend upward. In a wet state, the oxygen ions on the surface of Mn^II^–Fe^III^ dissolve in water and form hydroxide ions. The adhesion of hydroxide ions to the surface of Mn^II^–Fe^III^ leads to a decrease in hole concentration and causes the energy band to bend downward. Therefore, there is a difference in carrier concentration (hole concentration) between wet and dry areas, resulting in a potential difference. In addition, the ion solution has a higher electricity generation performance, which is attributed to the adsorption of more holes by chloride ions in the salt solution, further reducing holes in the wet zone, and thereby increasing the potential difference.

Multiple devices in series and parallel can be used to amplify energy output further to meet application requirements. As shown in Figure 5c, the voltage of one device is achieved up to 0.47 V, for three devices connected in series, it increases to ≈1.44 V, while the voltage of five devices connected in series increases to ≈2.3 V, exhibiting a linear increase behavior. Similarly, the current of one device rises to ≈40 µA, two devices in parallel increase to ≈73 µA, and the current of five devices in parallel increases to ≈100 µA (Figure 5d). Subsequently, the impact of different solutions on the energy output performance of Mn^II^–Fe^III^@CM was detected (Figure 5e). With water infiltrating into the Mn^II^–Fe^III^@CM, the voltage, and current output performance can achieve ≈0.46 V and ≈20 µA. When 2.5 wt% NaCl solution is infiltrated into Mn^II^–Fe^III^@CM, the voltage and current output increase to ≈0.48 V and ≈40 µA. As the NaCl concentration further increases to 3.5, 5, 10, 15, and 20 wt.%, the voltage output performance further improves to ≈0.49 V, ≈0.5 V, ≈0.51 V, ≈0.52 V, and ≈0.54 V, and the current output performance further improves to ≈59 µA, ≈62 µA, ≈63 µA, ≈67 µA, ≈70 µA, respectively. These results demonstrate that ions in solution play an important role in electrical energy output.

Figure 5f,g show the output voltages under ambient conditions with no solar irradiation. To fulfill application requirements, the output voltage of Mn^II^–Fe^III^@CM was increased by connecting several devices in series. As seen in Figure 5f, the voltage may be raised high enough and consistent to operate a calculator under ambient conditions without sunlight, indicating that Mn^II^–Fe^III^@CM has exceptional scaling characteristics. **Table** **1** represents the comparison of Mn and other 2D materials‐inspired solar evaporators for in situ solar evaporation and energy generation. The real‐time picture of output voltage obtained by the single device Mn^II^–Fe^III^@CM is shown in Figure 5g under ambient conditions. The aforementioned findings demonstrate that Mn^II^–Fe^III^@CM can perform continuous work via two distinct mechanisms: i) Utilizing solar evaporation and enhancing energy production during daylight, and ii) Generating energy under overcast conditions or at night. Furthermore, the voltage output of Mn^II^–Fe^III^@CM may be increased and stored to fulfill the needs of certain applications by linking many devices in a series configuration.

**Table 1:** Represents the comparison of Mn and other 2D materials‐inspired solar evaporators for in situ solar evaporation and energy generation.

Sr No#	Materials	Evaporation rate Kgm^−2^h^−1^	Output voltage V_out_ [mV]	Ref.
1	Ag/GO‐ PW@SiO_2_	1.09	25	[38]
2	PDMS‐CNT‐CNC	1.36	50	[13]
3	CNT filter paper	1.15	60	[39]
4	Carbonaceous Materials	1.36	95	[40]
5	PMD/MXene‐WCM	1.59	133.82	[41]
6	Fe_3_O_4_@PPy	1.98	199.5	[42]
7	MnO_2_@PPy nanocomposites	1.69	253	[43]
8	Mn^II^–Fe^III^@CM	2.39	480	This work

### 2.4 Photo‐Electrothermal Evaporation

**Figure 6:**
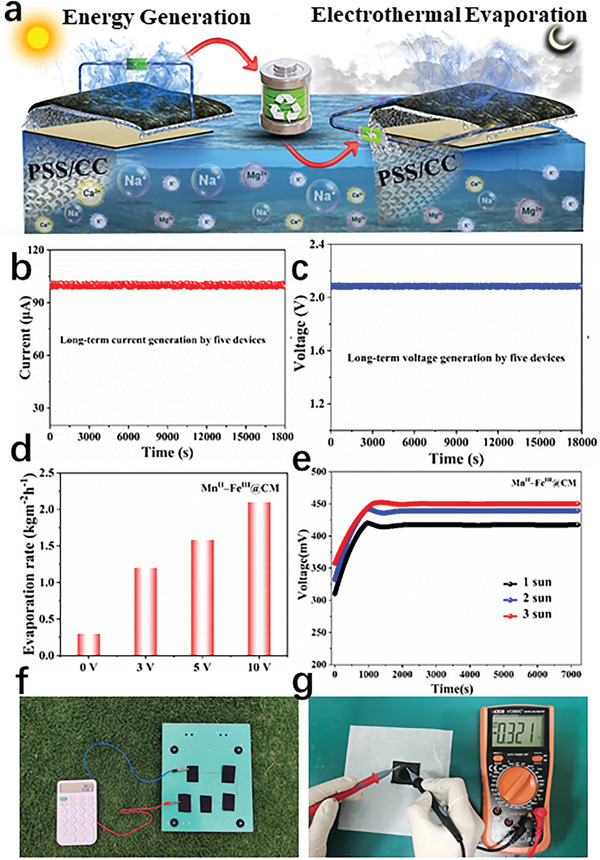
a) Schematic diagram of double‐sided evaporation system. b) Long‐term out current and c) voltage generation of Mn^II^–Fe^III^@CM of five devices connected in series. d) Electrothermal evaporation performance of Mn^II^–Fe^III^@CM. e) Output voltage performance of the Mn^II^–Fe^III^@CM under multiple solar intensities. f) Outdoor energy generation performance Mn^II^–Fe^III^@CM five devices connected in series enough to operate a calculator. g) The output voltage of single device Mn^II^–Fe^III^@CM.

Although solar photothermal evaporation systems have made good progress in recent years, it is still limited by the discontinuity of sunlight. The photothermal system lacks a heat source supply in the absence of light, and the interface temperature of the photothermal device rapidly decreases, making the system unable to exert effective evaporation performance. Combining the advantages of Mn^II^–Fe^III^@CM dual functionality, it is expected to solve the above problems through an energy management strategy. Based on the electricity generation performance of Mn^II^–Fe^III^@CM, if a suitable device is used to store electrical energy and then the energy is used for electrothermal vaporization, it will effectively solve the problem of low evaporation performance under dark conditions (**Figure** **6**a). Systematic experiments have revealed that Mn^II^–Fe^III^@CM not only outputs electrical energy in the absence of light but also can achieve full‐time functionality. To further verify that full‐time evaporation can be achieved through an energy management strategy, a DC power supply was used to heat the Mn^II^–Fe^III^@CM‐based evaporation system. Under the electric heating of 3, 5, and 10 V, the evaporation rate can reach 1.2, 1.58, and 2.1 kg m^−2^ h^−1^, respectively (Figure 6b). Further, the long‐term stability of the dual‐functional Mn^II^–Fe^III^@CM‐based evaporation was checked in terms of current and voltage generation as shown in Figure 6b,c. As obvious, a smooth output is obtained without any interruption throughout 6 h showing its tremendous potential for scalable applicability. The outdoor experiment was also performed and voltage under natural sunlight was calculated which was enough to run a calculator (Figure 6d). The outdoor investigations for Mn^II^–Fe^III^@CM‐based evaporation under natural solar irradiation and ambient temperature also carried out and recorded data is shown in Figures S10 and S11 (Supporting Information). For this, the experiment was performed on a sunny day (30 December 2024) from 8:00 a.m. until 6:00 p.m., the Hukseflux LP‐02 thermal pyrometer recorded sun intensity during the outdoor experiment. Solar flux, environmental temperature, evaporator surface temperature, and evaporation rate were recorded after each half an hour interval and the mean rate at which filtered water is produced is 1.2 mL m^−2^ h^−1^ and the evaporator surface temperature reached 29.2 °C under natural environment conditions. Hence, the Mn^II^–Fe^III^@CM evaporator prototype provides a design framework for the next advanced evaporator that will effectively meet the increasing need for fresh drinking water. Table S2 (Supporting Information) shows the comparison of evaporation performance of Mn^II^–Fe^III^@CM solar evaporator with the recently developed 2D and 3D solar evaporators. The energy management strategy can achieve full‐time evaporation for seawater desalination through sunlight and self‐sufficiency strategies. If the problem of energy storage is solved, its proposal will be beneficial for sustainable development, especially in remote areas.

## 3 Conclusion

In summary, viologen‐based 2D semiconductor compounds show potential for broadband photoresponse due to their strong cation‐π interactions, potentially increasing photoinduced electron transfer, and free radical product generation. Herein, the proposed strategy aims to achieve all‐weather utilization of viologen‐based 2D semiconductors anchored charcoal mask (Mn^II^–Fe^III^@CM) through dual‐function management. As an energy generator, the output voltage and current of Mn^II^–Fe^III^@CM can reach up to ≈480 mV and ≈60 µA cm^−2^ under ambient conditions. The maximum voltage can be further increased to ≈640 mV cm^−2^ under one sun illumination. In addition, Mn^II^–Fe^III^@CM is developed with a sided evaporation structure and controllable water transfer for high‐rate photothermal evaporation, offering a high evaporation rate of 2.39 kg m^−2^ h^−1^ under one sun (1 kW m^−2^) illumination. Furthermore, facing the problem of unsustainable sunlight during the evaporation process, storing electrical energy from Mn^II^–Fe^III^@CM through energy storage devices is expected to achieve all‐weather evaporation through electric heating. The study investigates Mn^II^–Fe^III^@CM for freshwater and electricity generation, providing a unique technology for saltwater desalination and offshore work platform energy access.

## 4 Experimental Section

### Materials

MnCl_2_·5H_2_O, and K_3_[Fe(CN)_6_] in AR grade were from were purchased from Shanghai Macklin Biochemical Co., LTD. MQCl·H_2_O (MQ^+^ = *N*‐methyl‐4,4′‐bipyridinium) was synthesized according to the same procedure reported in the literature.^[^ ^33^ ^]^ Charcoal mask, and carbon cloth, were purchased from Ningbo Mastertop Home Products Co., Ltd. All compounds maintained a purity level of 99% and were treated without the need for additional purification. MQCl·H_2_O (MQ^+^ = *N*‐methyl‐4,4′‐bipyridinium) was synthesized according to the same procedure reported in the literature.^[^ ^33^ ^]^

### Syntheses of [{Mn^II^(MQ)_2_}{Fe^III^(CN)_6_}]Cl·3H_2_O (1)

Typically, a 50 mL small beaker was placed in a 300 mL big one, which was filled with distilled water to approximately 0.5 cm above the top of the small beaker. A frozen 2 mL aqueous solution of MQCl·H_2_O (899 mg, 4 mmol) and MnCl_2_·5H_2_O (432 mg, 2 mmol) was thrown into the bottom of the small beaker, while the other frozen 2 mL aqueous solution of K_3_[Fe(CN)_6_] (659 mg, 2 mmol) was put into the bottom of the big beaker. The big beaker was sealed with plastic wrap and allowed to stand in the dark at room temperature for one week to yield dark brown cubic crystals. The crystals were filtered, washed with water and ethanol, and finally dried in air for 1 day. Yield based on K_3_[Fe(CN)_6_]: 40% for 1. All crystal samples for tests were carefully selected under a microscope. The synthesized viologen‐based 2D semiconductor crystals were ground and subsequently subjected to pyrolysis in a tube furnace at 400 °C for 2 hours, with an argon gas flow maintained at a ramp rate of 5 °C per minute. After the completion of the pyrolysis, the material was allowed to cool naturally to room temperature, resulting in the formation of Mn^II^–Fe^III^ powder.

### Fabrication of Mn^II^–Fe^III^@CM Solar Evaporator

A total of 0.2 g of dry Mn^II^–Fe^III^ powder and 0.1 g of sodium dodecyl sulfate (SDS), an anionic surfactant, were dispersed in 60 mL of deionized (DI) water to form a uniform dispersion (referred to as the Mn^II^–Fe^III^–SDS solution) through 1.5 hours of ultrasonic treatment. Similarly, 0.3 g of dry Mn^II^–Fe^III^ powder was dispersed in 60 mL of DI water, yielding another uniform dispersion (designated as the Mn^II^–Fe^III^ solution) after the same ultrasonic treatment. The rectangular charcoal mask (CM) substrate was initially immersed in the Mn^II^–Fe^III^ solution and dried in a convection oven at 80 °C following adequate soaking. This immersion and drying process was repeated three times to achieve a homogeneous Mn^II^–Fe^III^ precipitated fabric (Mn^II^–Fe^III^ @CM). Subsequently, the Mn^II^–Fe^III^ –SDS solution was deposited dropwise in the central region of the rectangular CM, totaling 15 mL added in increments of 5 mL, followed by drying at 80 °C. This deposition process was repeated four times. To enhance salt rejection performance, a side evaporation structure was designed, incorporating a cotton fabric treated with salt‐resistant polystyrene sodium sulfonate (PSS), enriched with sulfonate (SO_3_^–^) groups, and supported by polyethylene terephthalate (PET) foam for effective thermal management.

### Characterization

The single‐crystal X‐ray diffraction analyses of compounds are reported in the literature^[^ ^33^ ^]^ using a Rigaku SATURN70 CCD diffractometer with graphite monochromated Mo Kα radiation (λ = 0.71073 Å).^[^ ^33^ ^]^ The primitive structures were determined using the direct method, employing the Siemens SHELXTL Version 5 crystallographic software package.^[^ ^33^ ^]^ Field Emission Scanning Electron Microscopy (FESEM HITACH SU8220) was used to observe the morphologies of Mn^II^–Fe^III^@CM. X‐ray diffraction (XRD) data of Mn^II^–Fe^III^@CM was performed by a D8‐advance diffractometer (Bruker, Germany). THERMO FISHERSICENTIFIC Escalab 250Xi was used to test X‐ray photoelectron spectroscopy (XPS) data of Mn^II^–Fe^III^@CM.

### Water‐Driven Energy Generation

The electrical property data were tested by a data acquisition system (KEYSIGHT, DAQ970). The whole test environment was kept at 25–28 °C and 50–55% humidity.

### Solar Water Evaporation

Solar water evaporation tests were executed on an electronic balance with a solar simulator (CEL‐S500, AM1.5) to simulate sunlight. The light intensity was measured by using an optical power meter (SM206). Real‐time measurement of the water weight loss was recorded by a computer and evaporation system temperature was measured by thermocouples (OMEGA, TT‐K‐30‐SLE) and Infrared thermal imager (FLIR E5).

## Conflict of Interest

The authors declare no conflict of interest.

## Supporting information



Supporting Information

## Data Availability

The data that support the findings of this study are available from the corresponding author upon reasonable request.
