# Supplementary material for: Controlled Self‐Assembly and Photo‐Thermal Activation of Viologen‐Based 2D Semiconductors for Dual‐Function Energy Management in All‐Weather Applications
Source: Adv Sci (Weinh). 2025 Feb 8;12(13):2415101. doi: 10.1002/advs.202415101 (PMC11967839; doi:10.1002/advs.202415101)
Supplement: Supplementary file 1 — Supporting Information [file ADVS-12-2415101-s001.docx]

Supporting Information

Controlled Self-Assembly and Photo-Thermal Activation of Viologen-Based 2D Semiconductors for Dual-Function Energy Management in All-Weather Applications

Muhammad Sultan Irshad ^a,1^, Iftikhar Ahmed ^b,1^, Naila Arshad ^a^, Muneerah Alomar ^c^, Yue Shu ^a^, Guo Zhenzhen ^a^, Shafiq Ahmed ^d^, Ioannis Zuburtikudis ^e^, Shi Ruiqing ^f^, Tao Mei ^a^, Fan Xiaochao ^f,^*, Ho Xuan Nang^g^, Thuy-Duong Pham ^h^, Van-Duong Dao ^h,^*, Rong Li ^a,^*, Xianbao Wang ^a,^*

*^1^ Ministry-of-Education Key Laboratory for Green Preparation and Application of Functional Materials, School of New Energy and Electrical Engineering, Hubei University, Wuhan 430062, P.R. China.*

*^2^Environmental and Public Health Department, College of Health Sciences, Abu Dhabi University, P.O. Box 59911, Abu Dhabi, United Arab Emirates.*

*^3^State Key Laboratory of Advanced Technology for Materials Synthesis and Processing, School of Materials Science and Engineering, Wuhan University of Technology, Wuhan 430070, P.R. China.*

*^4^Department of Industrial Engineering, College of Engineering, King Saud University, Riyadh 11564, Saudi Arabia.*

*^5^Department of Chemical Engineering, Abu Dhabi University, P.O. Box 59911, Abu Dhabi, United Arab Emirates.*

*^6^Xinjiang Institute of Engineering, Urumqi, Xinjiang, 830047, China.*

*^7^Faculty of Vehicle and Energy Engineering, PHENIKAA University, Hanoi, Viet Nam*

*^8^Faculty of Biotechnology, Chemistry, and Environmental Engineering Phenikaa University Hanoi 100000, Viet Nam.*

*^9^School of Intelligent Manufacturing, Hubei University, Wuhan 430062, P.R. China.*

E-mail: duong.daovan@phenikaa-uni.edu.vn (Van-Duong Dao); rli@hubu.edu.cn (Rong Li); [fxc1979@xju.edu.cn](mailto:fxc1979@xju.edu.cn) (Fan Xiaochao); wxb@hubu.edu.cn (Xianbao Wang)





**Figure S1.** XRD spectra of viologen 2D semiconductor Mn^II^-Fe^III^ in crystal, powder, and 600 ^o^C.





**Figure S2.** Mn2p spectra of viologen 2D semiconductor Mn^II^-Fe^III^.





**Figure S3.** C1S spectra of viologen 2D semiconductor Mn^II^-Fe^III^.






**Figure S4.** Fe2p spectra of viologen 2D semiconductor Mn^II^-Fe^III^.

**Figure S5.** Comparative analysis of tensile stress-strain curve of Mn^II^-Fe^III^@CM.


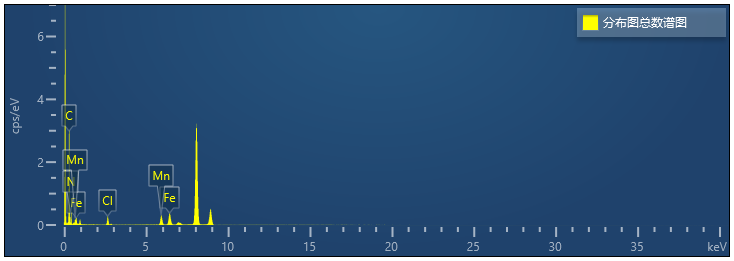
**Figure S6.** The total spectrum of distribution plots of viologen 2D semiconductor Mn^II^-Fe^III^.

**Table S1.** The total spectrum of distribution plots of viologen 2D semiconductor Mn^II^-Fe^III^.

| Element | k factor | Absorption  correction | Wt% | Wt% Sigma | At% |
| --- | --- | --- | --- | --- | --- |
| C | 2.738 | 1.00 | 65.61 | 0.43 | 80.00 |
| N | 3.476 | 1.00 | 13.14 | 0.40 | 13.74 |
| Cl | 1.017 | 1.00 | 4.33 | 0.11 | 1.79 |
| Mn | 1.165 | 1.00 | 7.71 | 0.17 | 2.06 |
| Fe | 1.165 | 1.00 | 9.20 | 0.19 | 2.41 |
| Total: |  |  | 100.00 |  | 100.00 |





**Figure S7.** UV absorption spectra of charcoal mask and Mn^II^-Fe^III^@CM.

**Figure S8.** Thermal conductivity of charcoal mask.





**Figure S9.** Thermal conductivity of charcoal mask.

**Note S1: Theoretical calculation section.**

**Calculations of the Heat Loss**:

The heat loss in the evaporation process mainly results from three aspects: heat radiation, heat convection, and heat conduction. The specific calculation methods were as follows^[1]^:

**Heat radiation**: The radiation loss could be calculated by Stefan–Boltzmann

$$\begin{aligned} Q_{rad}=\varepsilon\sigma\left( T_{1}^{4}-T_{2}^{4} \right) \left（ S1 \right） \end{aligned}$$

$$\begin{aligned} Radiation loss=\frac{Q_{rad}}{C_{opt}q_{i}}=\frac{\varepsilon\sigma\left( T_{1}^{4}-T_{2}^{4} \right)}{C_{opt}q_{i}} \left（ S2 \right） \end{aligned}$$

Where *𝜀* was the denotes emissivity (assuming a maximum emissivity of 1 for the evaporator), *𝜎* was the Stefan-Boltzmann constant (5.67×10^–8^ W m^–2^·K^4^), *T_1_* was the average surface temperature of the evaporator, and *T_2_* was the ambient temperature. Therefore, according to equations S1 and S2, we could calculate the radiation loss of the evaporator at 1 solar illumination.

**Heat convection**: Heat convection occurs between the surface of the solar absorber and the ambient environment. The radiation loss could be then calculated using Newton's law.

$$\begin{aligned} Q_{conv}=h\left( T_{1}-T_{2} \right) \left（ S3 \right） \end{aligned}$$

$$\begin{aligned} Convection loss=\frac{Q_{conv}}{C_{opt}q_{i}}=\frac{h\left( T_{1}-T_{2} \right)}{C_{opt}q_{i}} \left（ S4 \right） \end{aligned}$$

Where *Q* denoted the heat, *h* was the convection heat transfer coefficient (according to an early report, the convection heat transfer coefficient is about 5 W m^–2^ K^–1^).

**

Heat conduction**: The conduction loss here was the heat from the absorber to the bulk water. Due to the polystyrene insulation, there was negligible heat conduction loss of heat from the absorber to the bulk water accumulation.

**Figure S10.** Hourly records of the solar irradiation intensity, ambient temperature, and surface temperature of Mn^II^–Fe^III^@CM from 8:00 to 18:00.

**

**

**Figure S11.** Hourly records of the solar irradiation intensity and evaporation rate of Mn^II^–Fe^III^@CM from 8:00 to 18:00.

**Table S2.** Represents the detailed comparison of evaporation performances.

| Evaporator Material | Evaporation rate  (Kgm^-2^h^-1^) | Evaporator structure | Water to Vapor Conversion Efficiency | Water collection rate  (Kgm^-2^h^-1^) | Ref. |
| --- | --- | --- | --- | --- | --- |
| Carbonized cattail | 4.12 | 3D | 25% | 1.04 | ^[2]^ |
| Carbon black coated paper | 1.28 | 2D | 8% | 0.10 | ^[3]^ |
| PPy-coated SS mesh | 1.28 | 2D | 9% | 0.11 | ^[4]^ |
| 𝜆-Ti_3_O_5_-PVA hydrogel | 6.09 | 3D | 36% | 2.22 | ^[5]^ |
| CNT-PVA hydrogel | 2.63 | 3D | 65% | 1.72 | ^[6]^ |
| Black fabric | - | 2D | - | 0.31 | ^[7]^ |
| Printing paper | - | 2D | - | 0.25 | ^[8]^ |
| RGO foam | 2.4 | 2D | 42% | 1 | ^[9]^ |
| Black fabric | - | 2D | - | 0.12 | ^[10]^ |
| Graphene aerogels | 2.10 | 2D | 38% | 0.80 | ^[11]^ |
| Carbonized coconut fiber | 7.28 | 2D | 14% | 1 | ^[12]^ |
| Calcined melamine | 1.98 | 3D | 51% | 1 | ^[13]^ |
| PVA/PPy sponge | 2.03 | 3D | 31% | 0.62 | ^[14]^ |
| MXene aerogels | 1.46 | 3D | 51% | 0.75 | ^[15]^ |
| GO@SiO_2_ | 2.4 | 3D | 21% | 0.5 | ^[16]^ |
| PGO-agarose | - | 3D | - | 4.01 | ^[17]^ |
| Janus porous GO | 2.05 | 3D | 88% | 1.8 | ^[18]^ |
| Black fabric | - | 2D | - | 0.24 | ^[19]^ |
| CNT-coated fibers | - | 2D | - | 0.63 | ^[20]^ |
| Ni-MOF | 2.07 | 2D | 17% | 0.34 | ^[21]^ |
| ZCTP aerogel | 11.70 | 2D | 6% | 0.75 | ^[22]^ |
| TiNO_x_ membrane/PAM | 1.79 | 3D | 51% | 0.92 | ^[23]^ |
| PPy-paper folding | 2.25 | 3D | 94% | 2.11 | ^[24]^ |
| Carbon black/Al plate | - | 2D | - | 0.15 | ^[25]^ |
| CNPs/PAA hydrogel | - | 3D | - | 0.5 | ^[26]^ |
| RGO/LiCl aerogel | 4.0 | 3D | 53% | 2.1 | ^[27]^ |
| CuO/PVA, chitosan aerogel | 2.21 | 2D | 88% | 1.95 | ^[28]^ |
| TiNO_x_-coated Al plate | - | 2D | - | 1.06 | ^[29]^ |
| Black absorbent filler | - | 3D | - | 0.20 | ^[30]^ |
| Carbon black/PVA hydrogel | 3.7 | 2D | 65% | 2.4 | ^[31]^ |
| PPy coated melamine | 1.9 | 3D | 95% | 1.8 | ^[32]^ |
| Black cotton | - | 3D | 36% | 0.54 | ^[33]^ |
| Mn^II^–Fe^III^@CM | 2.80 | 3D | 92% | 1.2 | **This work** |

**References**

[1] M. S. Irshad, X. Wang, M. S. Abbasi, N. Arshad, Z. Chen, Z. Guo, L. Yu, J. Qian, J. You, T. Mei, *ACS Sustain. Chem. Eng.* **2021**, *9*, 3887.

[2] C. Zhang, B. Yuan, Y. Liang, L. Yang, L. Bai, H. Yang, D. Wei, W. Wang, H. Chen, *Sol. Energy Mater. Sol. Cells* **2021**, *227*, 111127.

[3] Z. Liu, H. Song, D. Ji, C. Li, A. Cheney, Y. Liu, N. Zhang, X. Zeng, B. Chen, J. Gao, others, *Glob. Challenges* **2017**, *1*, 1600003.

[4] L. Zhang, B. Tang, J. Wu, R. Li, P. Wang, *Adv. Mater.* **2015**, *27*, 4889.

[5] B. Yang, Z. Zhang, P. Liu, X. Fu, J. Wang, Y. Cao, R. Tang, X. Du, W. Chen, S. Li, *Nature* **2023**, 1.

[6] L. Wu, Z. Dong, Z. Cai, T. Ganapathy, N. X. Fang, C. Li, C. Yu, Y. Zhang, Y. Song, *Nat. Commun.* **2020**, *11*, 1.

[7] G. Ni, S. H. Zandavi, S. M. Javid, S. V Boriskina, T. A. Cooper, G. Chen, *Energy Environ. Sci.* **2018**, *11*, 1510.

[8] J. Xu, Z. Wang, C. Chang, B. Fu, P. Tao, C. Song, W. Shang, T. Deng, *Desalination* **2020**, *484*, 114423.

[9] H. Liang, Q. Liao, N. Chen, Y. Liang, G. Lv, P. Zhang, B. Lu, L. Qu, *Angew. Chemie* **2019**, *131*, 19217.

[10] F. A. Raihananda, E. Philander, A. F. Lauvandy, T. A. F. Soelaiman, B. A. Budiman, F. B. Juangsa, P. Sambegoro, *Results Eng.* **2021**, *12*, 100300.

[11] P. Zhang, Q. Liao, H. Yao, H. Cheng, Y. Huang, C. Yang, L. Jiang, L. Qu, *J. Mater. Chem. A* **2018**, *6*, 15303.

[12] Y. Yang, H. Feng, W. Que, Y. Qiu, Y. Li, L. Guo, Q. Li, *Adv. Funct. Mater.* **2023**, *33*, 2210972.

[13] F. F. Gong, H. Li, W. Wang, J. Huang, D. D. Xia, J. Liao, M. Wu, D. V Papavassiliou, *Nano Energy* **2019**, *58*, 322.

[14] Y. Shao, J. Tang, N. Li, T. Sun, L. Yang, D. Chen, H. Zhi, D. Wang, H. Liu, G. Xue, *EcoMat* **2020**, *2*, e12018.

[15] Q. Zhang, G. Yi, Z. Fu, H. Yu, S. Chen, X. Quan, *ACS Nano* **2019**, *13*, 13196.

[16] W. Jonhson, X. Xu, D. Zhang, W. T. Chua, Y. H. Tan, X. Liu, C. Guan, X. H. Tan, Y. Li, T. S. Herng, *ACS Appl. Mater. Interfaces* **2021**, *13*, 23220.

[17] X. Wu, T. Gao, C. Han, J. Xu, G. Owens, H. Xu, *Sci. Bull.* **2019**, *64*, 1625.

[18] T. Yang, H. Lin, K.-T. Lin, D. M. Saldarriaga, G. Yang, C. Guo, H. Zhang, J. Zhang, S. Fraser, A. K.-T. Lau, *Carbon N. Y.* **2022**, *199*, 469.

[19] S. Chen, P. Zhao, G. Xie, Y. Wei, Y. Lyu, Y. Zhang, T. Yan, T. Zhang, *Desalination* **2021**, *512*, 115133.

[20] K. Yang, T. Pan, S. Dang, Q. Gan, Y. Han, *Nat. Commun.* **2022**, *13*, 1.

[21] P. He, L. Hao, N. Liu, H. Bai, R. Niu, J. Gong, *Chem. Eng. J.* **2021**, *423*, 130268.

[22] S. Zhao, X. Zhang, G. Wei, Z. Su, *Chem. Eng. J.* **2023**, *458*.

[23] X. Mu, J. Zhou, P. Wang, H. Chen, T. Yang, S. Chen, L. Miao, T. Mori, *Energy Environ. Sci.* **2022**, *15*, 3388.

[24] W. Li, Z. Li, K. Bertelsmann, D. E. Fan, *Adv. Mater.* **2019**, *31*, 1900720.

[25] C. Du, C. Huang, *Appl. Therm. Eng.* **2022**, *201*, 117834.

[26] Z. Zhan, L. Chen, C. Wang, Y. Shuai, H. Duan, Z. Wang, *ACS Appl. Mater. Interfaces* **2023**, *15*, 8181.

[27] S. Dong, Y. Xu, C. Wang, C. Liu, J. Zhang, Y. Di, L. Yu, L. Dong, Z. Gan, *J. Mater. Chem. A* **2022**, *10*, 1885.

[28] H. Yao, P. Zhang, C. Yang, Q. Liao, X. Hao, Y. Huang, M. Zhang, X. Wang, T. Lin, H. Cheng, *Energy Environ. Sci.* **2021**, *14*, 5330.

[29] F. Wang, N. Xu, W. Zhao, L. Zhou, P. Zhu, X. Wang, B. Zhu, J. Zhu, *Joule* **2021**, *5*, 1602.

[30] Q. Wang, Z. Zhu, G. Wu, X. Zhang, H. Zheng, *Appl. Energy* **2018**, *224*, 510.

[31] H. Lu, W. Shi, F. Zhao, W. Zhang, P. Zhang, C. Zhao, G. Yu, *Adv. Funct. Mater.* **2021**, *31*, 2101036.

[32] W. Deng, T. Fan, Y. Li, *Nano Energy* **2022**, *92*, 106745.

[33] M. Mohsenzadeh, L. Aye, P. Christopher, *Energy Convers. Manag.* **2022**, *251*, 114902.
